# Influence of Solvent Composition and Surface Tension on the Signal Intensity of Amino Acids in Electrospray Ionization Mass Spectrometry

**DOI:** 10.5702/massspectrometry.A0077

**Published:** 2019-11-30

**Authors:** Ami Kageyama (Kaneshima), Akira Motoyama, Mitsuo Takayama

**Affiliations:** 1 Mass Spectrometry Laboratory, Graduate School of Nanobioscience, Yokohama City University, 22–2 Seto, Kanazawa-ku, Yokohama 236–0027, Japan

**Keywords:** ESI MS, amino acid, signal intensity, surface tension, solvent

## Abstract

The influence of solvent composition and surface tension on the signal intensity of deprotonated molecules [M−H]^−^ in electrospray ionization mass spectrometry (ESI MS) was evaluated using alanine (Ala), threonine (Thr) and phenylalanine (Phe), which have differing levels of hydrophobicity. The surface tension of the ESI solution was varied by changing the ratio of the organic solvents methanol (MeOH) and acetonitrile (MeCN) in water (H_2_O). In ESI MS, the signal intensity of all the amino acids was increased with decreasing surface tension for the two solutions, H_2_O/MeOH and H_2_O/MeCN. The use of H_2_O/MeCN was more favorable for achieving a strong signal for the analytes compared to H_2_O/MeOH. The smaller vaporization enthalpy of MeCN compared to MeOH was proposed as one of the most plausible explanation for this. The order of the signal intensity of amino acids was Phe>Thr>Ala, the same order as their hydrophobicity. It can be practically concluded that the use of solutions with lower surface tensions and lower vaporization enthalpies would result in higher signal intensities in ESI MS.

## INTRODUCTION

Electrospray ionization mass spectrometry (ESI MS) is widely used for the quantitative and qualitative analysis of various organic compounds, including amino acids, peptides, natural products and synthetic chemicals. When combined with liquid chromatography, a methodology known as liquid chromatography mass spectrometry, it can be exclusively used for the quantification of trace analytes, because of its extraordinary sensitivity and selectivity.^1–5)^ However, one of the major drawback of ESI MS is that it is strongly governed by the physicochemical properties of analytes and solvents, which makes it difficult to use as an absolute quantification method. A great number of studies have been reported in attempts to solve this issue and/or to understand the phenomena.^6–18)^

Regarding the solvent, Ikonomou *et al.* reported that an increase in volume ratio of methanol in a water/methanol solvent system increased the signal intensity of protonated cocaine in ESI.^8)^ Zhou and Hamburger also reported that the signal intensity of organic compounds was increased by increasing the volume ratio of organic solvent in water/methanol and water/acetonitrile systems.^9)^ They proposed that the increased signal intensity is due to the increase in efficiency of production of small droplets generated from the Taylor cone caused by the decrease in the surface tension of the solution system in ESI MS. The droplet size can be represented by the function of surface tension in a solvent as follows^10)^
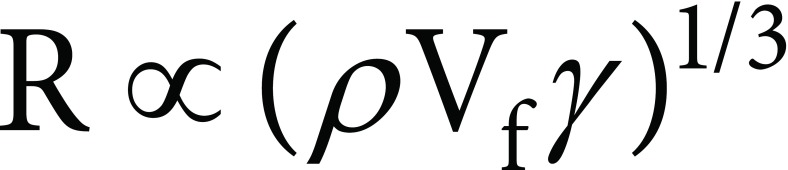
(1) where R, ρ, V_f_ and γ represent the droplet diameter, density, flow rate and surface tension of solvent, respectively.

The first plausible mechanism for gas-phase ion generation, which was reported by Kebarle and Verkerk,^11)^ consisted of major two steps after the generation of parent droplets from the Taylor cone. The two proposed steps are (a) shrinkage and fission of the charged droplets by the evaporation of solvent and (b) the production of gas-phase ions from the charged droplets. The process of repeated droplet fission of the parent droplets leads to formation of smaller progeny droplets. The fission of droplets occurs when the repulsion of the excess charge on the surface of the droplet overcomes the surface tension of the droplet. Gas-phase ions are produced from the charged droplets only when the droplet size is very small. Overall, it is obvious that the surface tension of parent droplets has a significant impact on the production of smaller droplets and thereby the ESI efficiency.

In addition to the surface tension of droplets, solvent pH is known to affect ESI signal intensity. For example, Liigand *et al.*^12)^ surveyed the influence of analyte p*K*_a_ and solvent pH on ESI signal intensity. Twenty-eight analytes having different p*K*_a_ and log *P* values were tested under acidic and neutral aqueous solvent conditions (pH 2.1–7.0). In general, the ionic dissociation of an analyte in a solution is determined by its p*K*_a_ and the solution pH. Acidic functional groups such as carboxylic acid groups are dissociated into a pair of negatively charged ions and one proton at a pH greater than their p*K*_a_, whereas basic amino groups are protonated at the pH values lower than their p*K*_a_. Such dissociation and protonation produce charged species which are less hydrophobic but will readily become gas-phase ions. To simplify the interpretation by cancelling the influence of solution pH and p*K*_a_ in this study, neutral amino acids, which are zwitterions of those functional groups, were used as model analytes. The pI values of the analytes are shown in Table 1. The net charge of those molecules becomes zero at a neutral pH, which allowed us to omit the difference in ionization efficiency among the analytes, and to avoid the use of pH modifiers.

**Table 1:** Table 1. Properties of amino acids used, molecular mass (*M*_r_), isoelectric point (pI), a measure of hydrophobicity (B&B, kJ/mol), gas-phase acidity (kJ/mol) and gas-phase basicity (kJ/mol).

Amino acid	*M*_r_	pI*^1^	B&B*^2^	GA*^3^	GB*^4^
Ala	89.1	6.00	+2.55	1430	864.1
Thr	119.1	5.60	+1.21	1388	886.3
Phe	165.2	5.48	−6.36	1418	888.0

*^1^ D. R. Lide. CRC Handbook of Chemistry and Physics. 89th Ed., CRC Press, Taylor & Francis Group, Boca Raton, FL, USA (2008), 7-1. *^2^ H. B. Bull and K. Breese. Surface tension of amino acid solutions: A hydrophobicity scale of the amino acid residues. *Archives of Biochem. Biophys.* 161: 665–670, 1974. *^3^ C. M. Jones, *et al*. Gas-phase acidities of the 20 protein amino acids. *Int. J. Mass Spectrom*. 267: 54–62, 2007. *^4^ A. G. Harrison. The gas-phase basicities and proton affinities of amino acids and peptides. *Mass Spectrom. Rev*. 16: 201–217, 1997.

It should be also noted that the nature of the hydrophobicity or surface activity of analytes can strongly affect signal intensity in ESI MS. Cech and Enke^13,14)^ and Tang and Kebarle^15,16)^ reported that more hydrophobic analytes have higher signal intensities in ESI MS, because such analytes tend to exist on the surface layer of ESI droplets. One of the authors reported that the signal intensity in ESI MS can be explained by dividing the total ion yield flux *J_i_* into two terms, namely ionization efficiency *I_i_* and vaporization flux *J*_v_,^17,18)^ as follows.
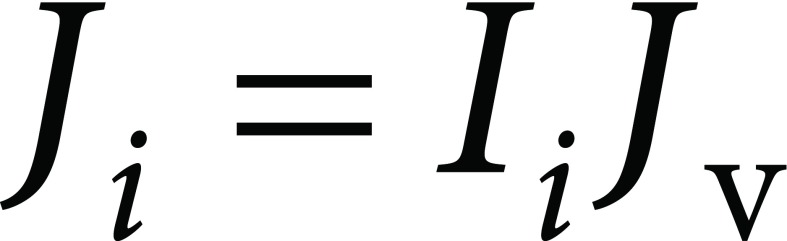
(2)

This equation means that the signal intensity is governed by the thermochemical natures of the analyte and solvent, such as gas-phase basicity and acidity for *I_i_*, and the physical properties of the analyte and solvent, such as hydrophobicity, surface tension and vaporization enthalpy for *J*_v_.

In this report, we examined the influence of the solvent composition and surface tension of the solution and the hydrophobicity of the analytes on the signal intensity in ESI MS. The purpose of this study was to empirically identify the influence of the hydrophobicity of analytes and surface tension of solutions on ESI signal intensity. As stated above, neutral amino acids, alanine (Ala), threonine (Thr) and phenylalanine (Phe), were chosen as model analytes. The ESI signal intensities were monitored in the negative-ion mode to probe the significance of surface activity. By design, the influence of other factors that may alter the ionization efficiencies *I_i_* were excluded because all of the test analytes share a common twitter ion structure. We anticipated that the interpretation of complex ESI ionization processes would be simplified by focusing on the difference in the non-ionic sidechains. To fully understand the processes, the surface tension of the solution was varied by changing the volume ratios of the organic solvent in water/methanol and water/acetonitrile systems.

## MATERIALS AND METHODS

### Reagents and Sample Preparation

Alanine, threonine and phenylalanine were purchased from the Peptide Institute (Minoh, Osaka, Japan). Acetonitrile, methanol and water were purchased from Wako Pure Chemical Industries, Ltd. (Osaka, Japan). All solvents were LCMS grade. All reagents were used without further purification. Each amino acid was dissolved in two solvent systems, water/acetonitrile and water/methanol at a 1 μM concentration. Each solvent was prepared at volume ratios of 0, 10, 30, 50, 70, 100%. The concentration of the analytes for surface tension measurements was 1 μM, representing typical concentrations used in ESI measurements.

### Mass spectrometry

Mass spectrometric measurements were performed on a LCMS-8050 triple-quadrupole mass spectrometer (Shimadzu, Kyoto, Japan) coupled with a Nexera HPLC system (Shimadzu, Kyoto, Japan). The instrument parameters were as follows: injection volume was 4 μL, flow rate of the flow-injection analysis solvent was 0.2 mL/min, the sample cooler temperature was 25°C, rate of N_2_ drying gas was 10 L/min, the rate of N_2_ nebulizing gas was 3 L/min, and the capillary voltage was −3.0 kV for the negative ion detection mode. The signal intensity was monitored for *m*/*z* values of deprotonated molecules [M−H]^−^ (*m*/*z* 88 for Ala, *m*/*z* 118 for Thr and *m*/*z* 164 for Phe) in the selected ion monitoring mode. The signal intensities were estimated from the peak areas of selected-ion chromatograms on flow injection analysis (triplicated measurements).

### Surface tension

The surface tensions of the bulk solutions with and without analytes were measured by the Wilhelmy plate method with a model DCAT 21 tensiometer (Dataphysics, Germany). Bulk solutions in 30 mL glass bottles were stored overnight in a thermostatic bath at 25°C. The value for the surface tension was estimated from the pulling force of the test solution when the bottom part of the plate barely touched the surface of the liquid. At this point, the liquid comes into contact with the plate, and the surface tension of the bulk solution acts along the periphery of the plate, attempting to pull the plate into the bulk solution. This pulling force was measured by a microbalance. One measurement required 5–10 min to reach surface tension equilibrium. The surface tension measurement was repeated 3 times per sample and the averaged values were used for evaluation.

## RESULTS AND DISCUSSION

### Influence of solvent composition on the signal intensity of amino acids

**Figure 1:**
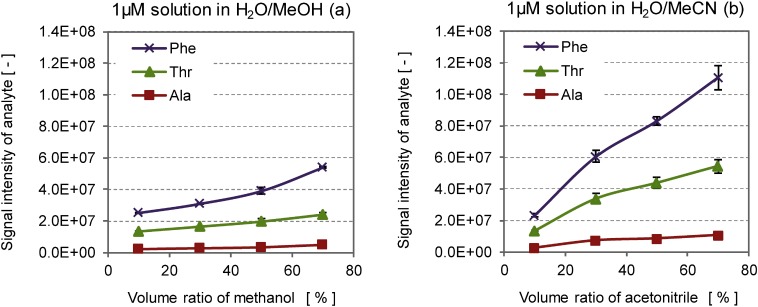
Fig. 1. ESI signal intensities for deprotonated molecules [M−H]^−^ of alanine, threonine and phenylalanine for two solvent systems of (a) H_2_O/MeOH and (b) H_2_O/MeCN.

Here we employed the negative-ion mode to estimate the signal intensity of amino acids, because the positive-ion mode in ESI MS frequently detects adduct cations such as [M+Na]^+^, [M+K]^+^ and [M+NH_4_]^+^, as well as [M+H]^+^. The signal intensities of the deprotonated amino acids [M−H]^−^ were obtained for two different solvent systems H_2_O/MeOH and H_2_O/MeCN, by changing the volume ratio of the organic solvent (Fig. 1). The volume ratio of organic solvent to water was varied from 10 to 70%. The relative standard deviations (RSD) of the signal intensity were in the range of 0.3–16%.

The RSD of the signal intensity were in the range of 0.3–16%.

The signal intensity of analytes increased with increasing volume ratio of the organic solvent in both solvent systems. In both solvent systems, the order of signal intensity of amino acids was Phe>Thr>Ala, which is consistent with the order of hydrophobicity, as indicated by the Bull and Breeze (B&B) hydrophobicity index,^19)^ as shown in Table 1. In addition, the use of H_2_O/MeCN as a solvent system resulted in a higher signal intensity than when H_2_O/MeOH was used. The results obtained above indicate that the signal intensity depends on the volume ratio of organic solvent, the organic solvent used and the hydrophobicity of the analyte.

We used the B&B hydrophobicity index in our investigations. The B&B hydrophobicity index is based on the measured surface tension values of each amino acid in a 0.1 M NaCl aqueous solution. It is known that the hydrophobicity value of the B&B index represents the surface activity of an analyte and has been used to discuss the hydrophobicity of amino acids. Although the octanol–water partition coefficient, log *P*, is also frequently used as an index of the hydrophobicity of amino acids, no apparent correlation with ESI signal intensities was found in the study in our system.

It should be noted that thermochemical properties of Ala, Thr and Phe, such as the isoelectric point (pI), gas-phase acidity (GA) and gas-phase basicity (GB) do not differ substantially from each other, as shown in Table 1. From this, it is reasonable to assume that the ionization (deprotonation) efficiencies *I_i_* in Eq. (2) for Ala, Thr and Phe are nearly equal to each other. Therefore, the difference in the signal intensity shown in Fig. 1 could be explained by the vaporization flux *J*_v_ in Eq. (2). As previously reported, both positive- and negative-ion yields of peptides can be positively correlated to the B&B hydrophobicity index.^17)^ This positive correlation between the signal intensity and analyte hydrophobicity can be explained by which analyte with a greater hydrophobicity is present in a higher concentration on the surface layer of the ESI droplets.^15,16,19)^ The analytes present on the surface layer are more likely to undergo vaporization than those located in the interior of the ESI droplets. The reason why the increase in the volume ratio of the organic solvent results in the increased signal intensity can be explained by the decreased surface tension of the solvents.^9)^ A solvent with a lower surface tension may result in smaller droplets, as is understood from Eq. (1). The formation of small droplets would allow the solvent and analyte molecules to evaporate from the surface of the ESI droplets.

The influence of droplet size on signal intensity was estimated by changing the flow rate. The flow-injection analysis of a 1 μM Ala solution using two solvent systems (10% and 70% MeCN) was conducted at different flow rates (0.05, 0.1, 0.2, 0.5 mL/min). The peak areas for Ala (from extracted ion chromatograms for *m*/*z* 88) decreased with increasing flow rate in both solvents (data not shown). Provided that the ion-transfer efficiency inside the vacuum region was constant at those flow rates, the smaller peak areas at higher flow rates would be explained by the difference in the sizes of ESI droplets.

Another important factor for the vaporization efficiency of the droplets is the vaporization enthalpy of the solvent used. The higher signal intensity in the use of H_2_O/MeCN solution compared to H_2_O/MeOH may be due to the lower vaporization enthalpy of acetonitrile compared to methanol, as shown in Table 2. For example, Wilhelm *et al.*^20)^ reported that solvents having high evaporation efficiencies produced small charged droplets and they attributed this to the solvent vaporization enthalpies. According to Fenn,^21)^ the enthalpy required to evaporate the solvent from the charged droplet was provided by the gas to which the charged droplets were exposed. Although the importance of enthalpy is obvious from the literature,^20,21)^ a more thorough investigation will be needed to reveal its significance on overall ESI processes.

**Table 2:** Table 2. Surface tension and vaporization enthalpy for the solvents used for ESI MS.

Solvent	Surface tension (mN/m)*^1^	Vaporization enthalpy (kJ/mol) *^2^
Water (H_2_O)	72.0	44.0
Methanol (CH_3_OH)	22.6	38.3
Acetonitrile (CH_3_CN)	30.0	33.8

*^1^ F. Charbonnier, C. Rolando, F. Saru, P. Hapiot, J. Pinson. Short time-scale observation of an electrospray current. *Rapid Commun. Mass Spectrom*. 7: 707–710, 1993. *^2^ J. S. Chickos, W. E. Acree, Jr. Enthalpies of vaporization of organic and organometallic compounds, 1880–2002. *J. Phys. Chem. Ref. Data* 32: 519, 2003.

### Influence of solvent composition and analyte on the surface tension of the solution

**Figure 2:**
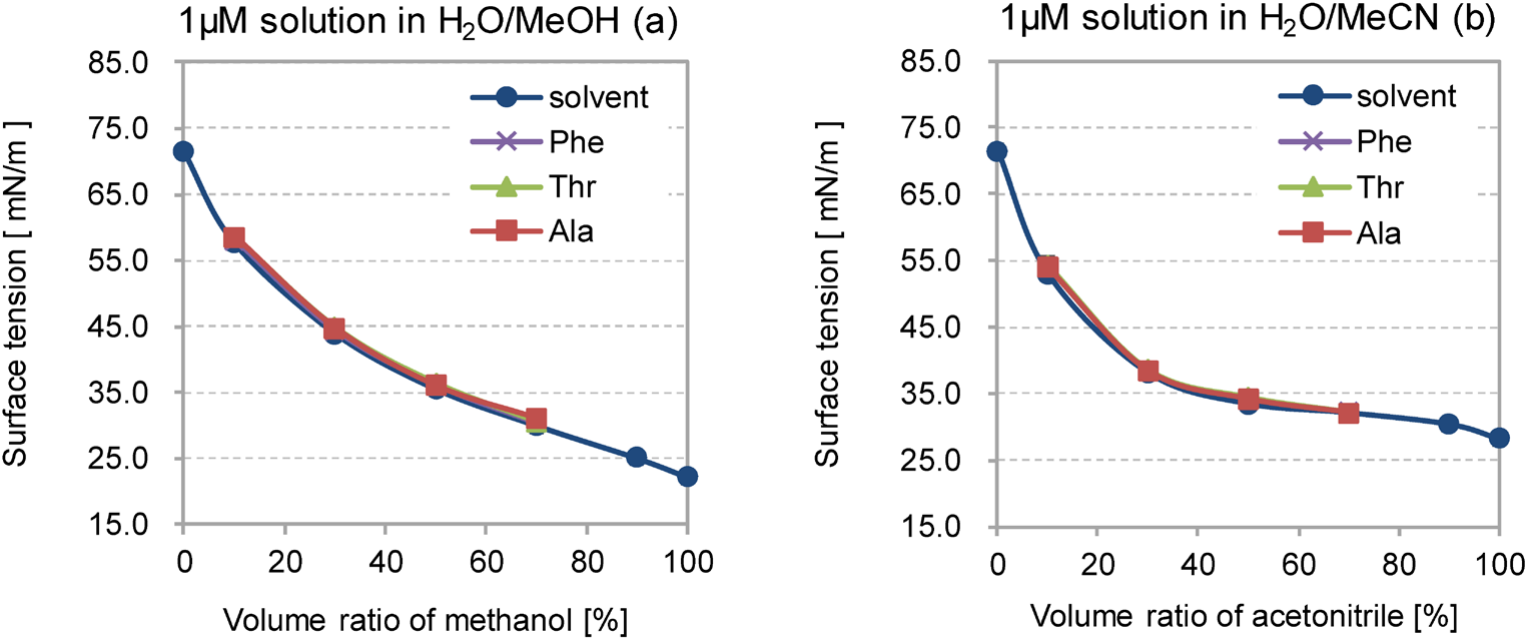
Fig. 2. Influence of the solvent composition of ESI solutions of (a) H_2_O/MeOH and (b) H_2_O/MeCN with and without amino acids. The RSD of the surface tension on triplicate analyses were less than 4%.

Figure 2 shows the influence of the volume ratio of organic solvent in two solution systems (H_2_O/MeOH and H_2_O/MeCN) on the surface tension of the solution with and without analytes. The surface tension values obtained in this study were consistent with previously reported findings.^22)^ The RSD of the surface tension on triplicate analyses were less than 4%. In both solution systems, the surface tension decreased steeply as the volume ratio of the organic solvent to water was increased from 10 to 40%, and the decrease in surface tension was less steep at organic solvent ratios in the 40–100% range.

The influence of analytes (Ala, Thr and Phe) on the surface tension of the solution was examined with an usual analyte concentration of 1 μM. All of the analytes had little effect on the surface tension at a concentration of 1 μM, as shown in Fig. 2. This may be due to the fact that the number of solvent molecules on the surface of the solution is much larger than that of analyte molecules. Assuming that 1 μM analyte molecules are uniformly dispersed in pure water, the number of water molecules is about 380 times greater than that of analyte molecules. This suggests that 1 μM amino acids do not greatly disrupt the hydrogen bonding network of the surface of the solutions used.

Interestingly, the surface tension of the H_2_O/MeCN mixture decreased more steeply with the ratio of organic solvent than that of H_2_O/MeOH at ratios of less than 50%, as shown in Fig. 2. Furthermore, the surface tension of H_2_O/MeCN was lower than that of H_2_O/MeOH when the ratios of organic solvent were under 50%. Regardless of the surface tension, the value for neat acetonitrile was lower than that of neat methanol (Table 2). This may be due to the endothermic and exothermic processes that occur from the mixing of an aprotic acetonitrile^23)^ and a protic methanol^24)^ with water, respectively. The endothermic property of the heat of mixing in the H_2_O/MeCN system suggests that acetonitrile molecules disrupt the hydrogen-bonding network of water molecules, while the exothermic property of H_2_O/MeOH allows it to form a stronger solvation network in the solution system. The disruption of the hydrogen-bonding network in water with acetonitrile may result in the decrease of the surface tension of the H_2_O/MeCN solution, which would be favorable for the evaporation of solvent and analyte molecules from the surface of the ESI droplets.

### Influence of the surface tension on the signal intensity

**Figure 3:**
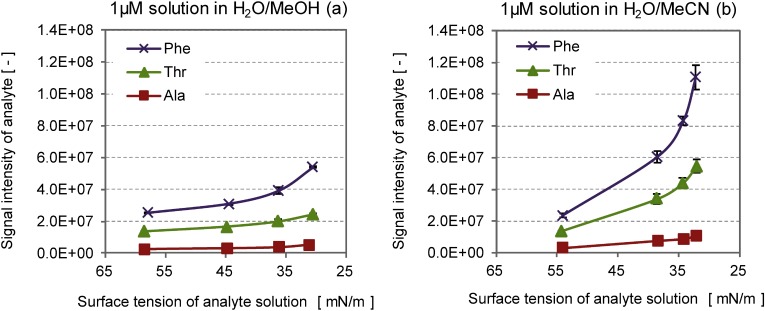
Fig. 3. ESI signal intensities of the deprotonated molecules [M−H]^−^ of alanine, threonine and phenylalanine against the surface tension of ESI solution systems of (a) H_2_O/MeOH and (b) H_2_O/MeCN.

As described above, it was suggested that the signal intensity is governed by several factors including the hydrophobicity of the analytes, the ratio of organic solvent to water (solvent composition), the heat of mixing protic (MeOH) and aprotic (MeCN) solvents, and the vaporization enthalpy of the solvents. The surface tension of the solution and the hydrophobicity of the analyte are particularly important properties governing the signal intensity, as shown in Fig. 3, which was prepared from data shown in Figs. 1 and 2. Figure 3 clearly shows that the signal intensity increases with decreasing surface tension of the solution and with the increase in hydrophobicity based on the B&B index of amino acids. In both solvent systems, the signal intensity of Ala was least affected by the reduction of the solvent’s surface tension. A plausible reason for this is its low B&B index (+2.55), which would force most of the Ala molecules to remain inside the solvent droplets in the tested solvent systems.

It is interesting to note that in Fig. 3 the signal intensity steeply increased with decreasing surface tension at values lower than 35 mN/m. The surface tension of 35 mN/m in the solution used here approximately corresponds to the ratio of organic solvents with a 50% or higher, as seen in Fig. 2. This indicates that the use of the ratio of 50% or higher acetonitrile composition to water is favorable for obtaining a higher signal intensity in a given analyte.

## CONCLUSION

The influence of several factors on the signal intensity of deprotonated amino acids [M−H]^−^ was evaluated using alanine (Ala), threonine (Thr) and phenylalanine (Phe) with differing levels of hydrophobicity and a common isoelectric point (pI) as model amino acids. The decrease in surface tension of two different solutions composed of H_2_O/MeOH and H_2_O/MeCN resulted in an increased signal intensity for all of the amino acids in both solution systems. The surface tension of the solution systems changed when the solvent composition, *i.e.*, the ratio of organic solvent to water, was changed. The use of H_2_O/MeCN as a solution was favorable for the signal intensity of the analytes used compared to H_2_O/MeOH, although the surface tension of neat acetonitrile was larger than that of neat methanol. The reason for why the use of H_2_O/MeCN is favorable for increasing the signal intensity may be due to the fact that the vaporization enthalpy of acetonitrile is smaller than that of methanol. In both solution systems, the order of the signal intensity of amino acids was in the same order of their hydrophobicity based on the B&B index, *i.e.*, Phe>Thr>Ala. Considering the common twitter ionic state, NH_3_^+^-Cα(-X)-COO^−^, and the pI values of the amino acids used (Table 1), the ion yields *J_i_* of deprotonated molecules [M−H]^−^ of Ala, Thr and Phe here appear to be governed by the vaporization flux *J*_v_ in the phenomenological Eq. (2). In fact, the factors described in this paper, such as the hydrophobicity of analytes, surface tension and the vaporization enthalpy of solvents, can be related to droplet formation and vaporization. It can be practically concluded, therefore, that the use of solutions with lower surface tensions and lower vaporization enthalpies would result in higher signal intensities for any given analyte when an ESI MS is being used.

## Notes

Mass Spectrom (Tokyo) 2019; 8(1): A0077
